# Maternal bonding styles in smokers and non-smokers: a comparative study

**DOI:** 10.1186/s12991-016-0118-y

**Published:** 2016-11-11

**Authors:** Iren Csala, Monika Elemery, Fruzsina Martinovszky, Peter Dome, Balazs Dome, Gabor Faludi, Imola Sandor, Zsuzsa Gyorffy, Emma Birkas, Judit Lazary

**Affiliations:** 1 Department of Psychiatry and Psychotherapy, Semmelweis University, Budapest, Hungary; 2 Institute of Behavioral Sciences, Semmelweis University, Budapest, Hungary; 3 National Institute of Psychiatry and Addiction, Budapest, Hungary; 4 Department of Tumor Biology, National Koranyi Institute of Pulmonology, Budapest, Hungary; 5 Department of Thoracic Surgery, Medical University of Vienna, Vienna, Austria; 6 Department of Thoracic Surgery, National Institute of Oncology and Semmelweis University, Budapest, Hungary; 7 Division of Molecular and Gender Imaging, Department of Biomedical Imaging and Image-guided Therapy, Medical University of Vienna, Vienna, Austria

**Keywords:** Parental bonding style, Nicotine dependence, Smoking onset, Depression

## Abstract

**Background:**

Parental bonding has been implicated in smoking behavior, and the quality of maternal bonding (MB) has been associated with poor mental health and substance use. However, little is known about the association of MB and the smoking of the offspring.

**Methods:**

In our study, 129 smokers and 610 non-smoker medical students completed the parental bonding instrument, which measures MB along two dimensions: care and overprotection. Four categories can be created by high and low scores on care and overprotection: optimal parenting (OP; high care/low overprotection); affectionless control (ALC; low care/high overprotection); affectionate constraint (AC; high care/high overprotection), and neglectful parenting (NP; low care/low overprotection). Nicotine dependence was assessed by the Fagerstrom Nicotine Dependence Test, exhaled CO level, and daily cigarette consumption (CPD).

**Results:**

Higher CPD was significantly associated with lower overprotection (*p* = 0.016) and higher care (*p* = 0.023) scores. The odds for being a smoker were significantly higher in the neglectful maternal bonding style compared to the other rearing styles (*p* = 0.022). Besides, smokers showed significantly higher care and lower overprotection scores with the Mann–Whitney U-test than non-smokers, although these associations did not remain significant in multiple regression models.

**Conclusion:**

Our results indicate that focusing on early life relationship between patient and mother can be important in psychotherapeutic interventions for smoking.

*Registration trials* retrospectively registered

## Background

Smoking is the leading cause of premature death, preventable morbidity, and disability worldwide [1–3]. Despite the huge effort for decreasing the health consequences of smoking, it is still an unsolved problem. In order to reduce smoking initiation and to conduct an efficient and successful quitting therapy, it is important to know the psychosocial risk factors for smoking initiation, nicotine dependence, and failing to quit. Earlier studies have identified several of those factors such as low socioeconomic status, low educational level, peer smoking, and family influences [4–6].

The majority of studies on the effect of the smoker’s parents on their tobacco use focused on the parents’ smoking behavior, the parent’s beneficial attitudes toward smoking, and parental practices against smoking. Those studies found that all these factors are related to the offspring’s smoking outcomes [5–7]. Less is known about the effect of parental bonding and attachment, and even less about the effect of maternal parenting style.

Adult smoking often starts at the adolescent age, and almost all adult smokers have their first smoking experiment by the age of 16 [8, 9]. By the age of 18, they are regular smokers already [10]. Therefore, the experience of the first 16 years, including the parents’ influence such as parental bonding as the parental bonding instrument measures it, is suggested to play an important role in the development of smoking behavior.

There is a large body of evidence suggesting that parental warmth, closeness, acceptance, emotional support, and emotional availability are associated with decreased chance of smoking or substance use [11–16]. However, the association between parental control and smoking behavior is inconsistent in the literature. Most studies reported that parental behavior control and parental monitoring relate inversely to smoking [12, 14, 17], but not all studies could confirm these findings [13, 15, 18]. Other studies even found a direct association between substance use and parental control [19, 20]. There are studies which distinguish strict control from psychological control and emphasize that the quality of control is determinant, i.e., while moderate and consistent strict control is beneficial, psychological control does not serve the offspring’s healthy mental development [18, 21]. Another aspect of parenting was also investigated, namely encouraged autonomy by the parents which partially faces the psychological control, and it was also suggested to be beneficial against smoking initiation and related to better quitting outcomes [22].

The results described above propound that there might be an optimal combination of parenting techniques and there might be a worst case scenario for smoking development. The literature confirms this expectation: authoritative parenting style, which is labeled as high warmth and high control from the parents, has been documented consistently to show the best substance use and smoking outcomes [23–27]. On the other hand, parental neglect, the combination of low care and low control has been found to be the greatest risk of smoking among examined parenting styles [21, 23, 27].

As regards maternal bonding, there are only sporadic results. Most studies only investigated parental rearing style, and only a few of them examined the effect of maternal bonding separately. The results of these studies are inconsistent. Some studies reported an inverse relation between high maternal care and smoking or substance use [15, 27, 28], while other researchers have not found such a correspondence [13]. In addition, the degree of maternal control has been implicated in substance use including smoking, but the direction of the relationship is not clear [19, 27].

Thus, the main purpose of the current study was to reveal and compare the maternal bonding styles in samples of smokers and non-smokers.

## Methods

### Study subjects

A dataset of 831 subjects was examined in our study, including 221 treatment-seeker smokers (112 males and 108 females with mean age of 51.2 ± 12.4 years) from 5 Hungarian quitting centers and 610 non-smoker medical students (198 males and 610 females with mean age of 22.4 ± 2.1).

The control non-smoker volunteers were all medical student volunteers (198 males and 412 females) from the medical faculties of the four medical universities in Hungary: Semmelweis University (Budapest), and Universities of Pécs, Debrecen, and Szeged. The mean age of this group was 22.4 ± 2.1. In order to avoid bias resulting from the educational differences between the two subgroups, only those smoker individuals were selected for this analysis who had high school graduation or degree. This moderately or highly qualified smoker group consists of 129 individuals (61 males and 68 females), with a mean age of 52.4 ± 12.8.

The difference in the mean age of smokers and non-smoker controls in our study sample is notable. However, it has no effect on the individuals’ perception of their mother’s behavior as it is measured by the PBI, since it is proven to be stable in time [29]. Besides, according to the literature, the mean age of smoking initiation is below 18 years [10], which suggests that non-smokers of the medical students will not become a smoker later.

Smoker participants were adult tobacco users who were committed to quitting. This study presents the data of their pre-quitting, first examination. The control group consisted of 610 psychiatrically healthy non-smoker medical student volunteers. Smoking was confirmed or excluded based on the scores of the Fagerstrom Test for Nicotine Dependence and daily cigarette consumption.

### Measures

#### Smoking variables

Nicotine dependence was assessed using the Fagerstrom Test for Nicotine Dependence (FTND), a widely used and validated 6-item measurement scoring from 0 to 10 [30]. Besides, the average daily number of cigarettes (cigarettes per day, CPD) and the exhaled carbon monoxide level (CO) were also obtained.

Treatment-seeking smoker participants were included in this study if all of the following criteria were fulfilled: above four points of FTND, above 10 ppm CO concentration in exhaled air, and at least 10 smoked cigarettes per day in the last month. The heavy smoker subgroup (HS) was defined as smokers with a daily consumption of over 20 cigarettes, and the light smoker subgroup (LS) was defined as those with a daily consumption of 20 or below, which is a common criteria of the intensity of smoking in the literature [31, 32].

Participants were selected into the control group only if they did not fit the described smoking criteria.

#### Parental bonding instrument (PBI)

The maternal version of the parental bonding instrument (PBI) [33, 34] was used to explore the individuals’ perceived maternal bonding patterns. This 25-item self-report questionnaire examines retrospectively the maternal rearing style from the subject’s view in their first 16 years on a 4-point Likert scale scoring from 0 to 3. In this study, we used only the maternal part of the instrument, where each item is a statement about the mother’s attitude and behavior. The maternal PBI measures the maternal bonding style along two dimensions: care (13 items) and overprotection (12 items). Both dimensions have two poles: maternal care is defined by emotional warmth, affection, trust, empathy, and closeness (high scores) or emotional coldness, neglect, and rejection (low scores), while maternal overprotection is characterized by the discouragement of autonomy and independence, excessive control, and intrusion (high scores) or reassuring independence and autonomy (low scores). Values from these dimensions can be used separately and can be divided into high and low scores according to the defined cut-off points: 13.5 points for maternal overprotection (high overprotection: HOP; low overprotection: LOP) and 27.0 points for maternal care (high care: HC; low care: LC).

The subgroups of the care and the overprotection scales can be combined by creating four specific maternal rearing styles: optimal parenting (OP; high care and low overprotection), affectionless control (ALC; low care and high overprotection), neglectful parenting (NP; low care and low overprotection), and affectionate constraint (AC; high care and high overprotection).

Mood and age only have a slight effect on the perception of parenting assessed by the PBI, since it is stable across time [29, 35].

#### Statistical analysis

The Kolmogorov–Smirnov test was performed to analyze the distribution of our variables. The differences of the PBI variables in the smoker and non-smoker groups were compared with the Mann–Whitney U-test and binary logistic regression. Besides these methods, linear regression was also used for revealing the association between continuous smoking and PBI variables. All regression analyses were adjusted for age and gender, except for the gender analyses, which were only adjusted for age. Besides, the gender differences in the frequency of the four PBI categories were tested with Chi-square test. Data were analyzed using SPSS version 20.0 software (IBM corp.) and are presented as mean (M) ± standard deviation (SD). Significance level was set at *p* < 0.05.

## Results

### Characteristics of the studied population

A sample of 729 individuals participated in this study including 129 smokers (61 males and 68 females) and 610 non-smoker volunteers (412 females and 198 males), with a mean age of 52.4 ± 12.8 and of 22.4 ± 2.1, respectively.

Among smokers, the mean value of FTND was 6.4 ± 1.2, the average daily number of cigarettes smoked was 21.0 ± 7.1, and exhaled CO concentration was 18.6 ± 7.6 ppm. The ratio of heavy smokers was significantly higher among male smokers (66.7%) compared to that among female smokers (47.1%; *p* = 0.020). No other gender differences were found regarding the smoking features (Table 1).

**Table 1:** Smoking characteristics of the study population

Smoking properties	Total	Males	Females
FTND	6.4 ± 1.2	6.4 ± 1.2	6.4 ± 1.1
CO level (ppm)	18.6 ± 7.6	18.9 ± 7.5	18.5 ± 7.8
CPD	21.0 ± 7.1	21.8 ± 7.1	20.3 ± 7.2
HS (≥20 CPD)	55.8%	66.7%	47.1%^a^

^a^ Statistically significant difference between males and females, ^a^<0.01

*FTND* Fagerstrom Nicotine Dependence Test; *CPD* cigarette per day; *HS* heavy smoker

The average care and overprotection scores were 29.7 ± 6.6 and 13.1 ± 7.8 in the total sample, respectively. The most frequent maternal bonding subtype in the total population was the optimal parenting subtype (50.1%) followed by the affectionate constraint (25.4%), the affectionless control (17.1%), and the neglectful parenting (7.4%) subtypes. Several gender differences were found in PBI variables. First, the care score was significantly higher among males than among females, but only in the smoker subgroup (Care_males_ = 28.9 ± 6.2, Care_females_ = 25.3 ± 8.7, *p* = 0.011).

The ratio of affectionate constraint was slightly higher among males in the total sample (AC_males_ = 30.2%, AC_females_ = 22.9%, *p* = 0.019) and also in the non-smoker cohort (AC_males_ = 32.8%, AC_females_ = 24.0%, *p* = 0.025). In the smoker group, optimal parenting and high care showed significantly higher proportion among males (OP_males_ = 48.3%, OP_females_ = 29.4%, *p* = 0.022; HC_males_ = 70.0%, HC_females_ = 45.6%, *p* = 0.004), while affectionless control and high overprotection were more frequent among females (ALC_males_ = 15.0%, ALC_females_ = 38.2%, *p* = 0.003; HOP_males_ = 36.7%, HOP_females_ = 54.4%, *p* = 0.033) (Table 2).

**Table 2:** Demographic and maternal bonding characteristics of the study population

	Smokers			Non-smokers		
	Total	Males	Females	Total	Males	Females
N	129	61 (47.3%)	68 (52.7%)	610	198 (32.5%)	412 (67.5%)
Age (M ± SD)	52.4 ± 12.8	52.0 ± 14.2	52.8 ± 11.4	22.4 ± 2.1	22.5 ± 2.3	22.3 ± 2.1
*MB*						
Care (M ± SD)	27.0 ± 7.7	28.9 ± 6.2	25.3 ± 8.7^b^	30.3 ± 6.2	30.3 ± 5.4	30.2 ± 6.6
Protection (M ± SD)	14.6 ± 8.6	13.2 ± 8.1	15.8 ± 8.9	12.8 ± 7.6	13.4 ± 7.3	12.6 ± 7.7
AC	18.6%	21.7%	16.2%	26.9%	32.8%	24.0%^b^
OP	38.8%	48.3%	29.4%^b^	52.5%	50.0%	53.6%
ALC	27.1%	15.0%	38.2%^b^	14.9%	13.6%	15.5%
NP	15.5%	15.0%	16.2%	5.7%	3.5%	6.8%
HC/LC	57.4%/42.6%	70.0%/30.0%	45.6%/54.4%^a^	79.3%/20.7%	82.8%/17.2%	77.7%/22.3%
HOP/LOP	45.7%/54.3%	36.7%/63.3%	54.4%/45.6%^b^	41.8%/58.2%	46.5%/53.5%	39.6%/60.4%

^a,b^ Statistically significant difference between males and females, ^a^<0.01, ^b^<0.05)

*MB* maternal bonding; *AC* affectionate constraint; *OP* optimal parenting; *ALC* affectionless control; *NP* neglectful parenting; *HC* high care; *LC* low care; *HOP* high overprotection; *LOP* low overprotection; *M* mean; *SD* standard deviation

### The effect of maternal bonding on smoking variables

The 129 treatment-seeker smokers were included in an association analysis of maternal bonding and smoking behavior.

First, we analyzed the smoking variables in the four maternal bonding subtypes and obtained no significant differences between the maternal bonding subtypes regarding the FTND score and the CO level. Similarly, care and overprotection scores had no effect on any of the latter smoking variables, as categorical variables of care and overprotection scores (high care, low care, high overprotection, and low overprotection) were not associated with the FTND score and the CO level either. As regards the daily cigarette consumption, higher maternal Care was associated with reduced CPD (*p* = 0.050), and higher maternal overprotection was associated with increased CPD in total sample (*p* = 0.016). Besides, the average CPD was significantly higher in the low care subgroup than in the high-care subgroup, but only among females (CPD_LC_ = 21.8, CPD_HC_ = 20.2, *p* = 0.014). We also found that in the low care subgroup, the odds of being a heavy smoker were significantly higher compared to those in the high-care subgroup in the total sample (*p* = 0.050, Exp(B) = 2.2) and a similar difference appeared within the female smoker cohort (*p* = 0.021, Exp(B) = 3.4).

### Differences of maternal care and overprotection between smokers and non-smokers

We compared the maternal bonding scales and subscales of the PBI between treatment-seeker smokers and non-smokers. Basic data of the PBI variables for each separate group, including gender subgroups, are shown in Table 2.

First, comparison tests of continuous variables were performed with the Mann–Whitney U-test in the total sample and also in gender subgroups.

Care score was significantly higher among non-smokers in the total sample (*p* < 0.001) and among females (*p* < 0.001), while among males this association showed only marginal significance (*p* = 0.066). As regards maternal overprotection, smokers had significantly higher scores on this scale, but only among females (*p* = 0.005).

After running these statistical analyses, we also tested the differences of care and overprotection scores between smokers and non-smokers with binary logistic regression adjusted for age and gender. In this case, significant association was not obtained in the total sample, not even in gender subgroups.

Binomial variables of the care and overprotection scales did not significantly differ either between smoker and non-smoker individuals.

### The four categories of the PBI in smokers and non-smokers

Exploring the association between smoking and maternal bonding, the distribution of the four maternal bonding subtypes were examined separately in smokers and in non-smokers with binary logistic regression adjusted for age and gender. Detailed data about the distribution of maternal bonding subtypes in each group are presented in Table 2.

Only one maternal bonding subtype showed significant association with smoking: the neglectful parenting style, which is defined as the combination of low care and low overprotection of the mother. The odds for being a smoker were significantly higher among individuals who perceived neglectful parenting from their mothers (Exp(B) = 32.5, *p* = 0.020).

Within the affectionate constraint, the optimal parenting and the affectionless control subgroups, no significant differences were detected between smokers and non-smokers.

## Discussion

Our findings confirmed that neglectful maternal bonding style (low care and low overprotection) has an important effect on whether an individual becomes a smoker, but it has no effect on smoking quantity or the level of nicotine dependence.

In the literature, most of the studies on the relationship between parental bonding and smoking did not separate the behavior of the mother and the father. Foxcroft et al. investigated adolescents (between the ages of 12 and 16) in ‘neglecting families’ and found that the ratio of adolescent smokers was higher in these types of families [21]. Similar results were reported by Chassin et al. about adolescents from families with low control and acceptance, which is very similar to the definition of neglectful parenting, showing a higher rate of smoking initiation [25]. In line with these results, Adalbjarnardottir et al. found that adolescents’ experimentation with smoking at age of 14 was more frequent among adolescents of neglectful parents [24].

A recent large-sample study among adolescents in China conducted by Wang et al. investigated the effect of maternal and paternal bonding, separately on smoking. They reported that maternal neglect was strongly related to higher odds for current smoking, while paternal neglect did not show association with current smoking [27].

These results suggest that maternal neglectful parenting might have an effect on experimentation and early stage of smoking initiation, and on the intensity of smoking. Besides, it might be more relevant in the development of smoking than paternal neglectful parenting.

The underlying biological mechanism of the connection between smoking and maternal neglect might be associated with the dopaminergic system [36]. There is some evidence that the quality of maternal attachment has an important effect on the development of the dopaminergic pathways, which plays a crucial role in nicotine dependence [37, 38] and in regulating maternal behavior as well [39]. An animal study by Meaney et al. examined rat pups after prolonged maternal separation and found that later, when these animals were already adult animals, these animals showed increased behavioral sensitivity to cocaine, which causes dopamine release in mesocorticolimbic dopaminergic neurons, suggesting that prolonged maternal separation is connected to higher susceptibility to addiction later in life through the altered development of the mesocorticolimbic dopaminergic system [40]. It confirms our assumption that maternal neglectful parenting has an important role in the development of smoking.

The perceived maternal bonding predicts the later maternal behavior of the female offspring [36, 41], which means that the maternal attachment style could transmit to the next generation causing a persistent cyclic problem in ‘neglectful families.’

In our study, childhood experience of maternal care and overprotection was associated with daily cigarette consumption. No data in the literature were found about the relationship between maternal bonding and the intensity of smoking.

Our findings on the effect of maternal care and overprotection on smoking behavior were not convincing as it did not remain significant after adjusting the test for age and gender. However, based on the literature, maternal care and overprotection are related to smoking [15, 27]. Probably, the notable difference in age between smoker and non-smoker subgroups of our study accounted for the confounded results. Further investigations are required to clarify this discrepancy.

The early relation to the mother seems essential in later mental health. The lack of love, warmth, care, and affection and the complete absence of control and protection at the same time might be the most harmful maternal rearing style. However, it is a perceived maternal bonding, which not necessarily reflects the real behavior of the mother.

There are several limitations to our study. First, the non-smoker subgroup consists of only medical students, which causes notable differences in age and occupation from the smoker subgroup. Besides, the smoker subgroup has only individuals with high school graduation or degree. The low sample size of the smoker subgroup is also an important limitation to our study.

## Conclusion

In conclusion, our data confirmed the importance of the maternal behavior in the development of smoking. Therefore, focusing on the early life relationship between the patient and his/her mother can be helpful in psychotherapy. As negative parenting behavior has a pathological effect during the early age on the onset of smoking in the adolescence, the education of parents about dysfunctional attitude can be an additional element of the preventive programs in the mental health systems.

## Abbreviations

AC: affectionate constraint
ALC: affectionless control
CO: carbon monoxide
CPD: cigarettes per day
FTND: Fagerstrom Test for Nicotine Dependence
HC: high care
HOP: high overprotection
HS: heavy smoker
LC: low care
LOP: low overprotection
LS: light smoker
M: mean
MB: maternal bonding
NS: non-smoker
NP: neglectful parenting
OP: optimal parenting
PBI: parental bonding instrument
S: smoker
SD: standard deviation
